# Clusterin Silencing in Prostate Cancer Induces Matrix Metalloproteinases by an NF-*κ*B-Dependent Mechanism

**DOI:** 10.1155/2019/4081624

**Published:** 2019-12-06

**Authors:** Martina Bonacini, Aide Negri, Pierpaola Davalli, Valeria Naponelli, Ileana Ramazzina, Chiara Lenzi, Saverio Bettuzzi, Federica Rizzi

**Affiliations:** ^1^Unit of Clinical Immunology, Allergy and Advanced Biotechnologies, Azienda Unità Sanitaria Locale-IRCCS di Reggio Emilia, Reggio Emilia 42122, Italy; ^2^Department of Medicine and Surgery, University of Parma, Parma 43126, Italy; ^3^Department of Biomedical, Metabolic and Neural Sciences, University of Modena and Reggio Emilia, Modena 41125, Italy; ^4^Centre for Molecular and Translational Oncology (COMT), University of Parma, Parma 43124, Italy; ^5^National Institute of Biostructure and Biosystems (INBB), Viale Medaglie d'Oro, Rome 00136, Italy

## Abstract

Clusterin (CLU) is a stress-activated glycoprotein, whose expression is altered both in inflammation and cancer. Previously, we showed that abrogation of CLU expression in cancer-prone mice (TRAMP) results in the enhancement of tumor spreading and homing, concomitant with an enhanced expression of NF-*κ*B. In the present paper, we carried out an extensive experimental work by utilizing microarray gene expression data, as well as *in vitro* and *in vivo* models of prostate cancer (PCa). Our results demonstrated that (i) CLU expression is significantly downregulated in human PCa and inversely correlates with the expression of p65 in metastases; (ii) CLU overexpression in PCa cells reduces the Ser_536_ phosphorylation of p65, inhibits NF-*κ*B nuclear translocation, and reduces the transcription of matrix metalloproteinase-9 and metalloproteinase-2 (MMP-9 and MMP-2). Conversely, CLU silencing promotes NF-*κ*B activation and transcriptional upregulation of MMP-9; and (iii) expression and activity of MMP-2 and MMP-9 are increased in CLU^−/−^ mice (CLUKO) and in TRAMP/CLUKO mice in comparison to their relative Clu^+/+^ littermates. Taken together, our data support the hypothesis that CLU downregulation, an early and relevant event in PCa onset, may inhibit NF-*κ*B activation and limit the execution of a transcriptional program that favor the disease progression towards a metastatic stage.

## 1. Introduction

Chronic infection and inflammation have been recognized to start cell transformation and promote cancer development [1]. The transcription factor NF-*κ*B is the main trigger of proinflammatory processes and key molecular link of inflammation to tumor initiation and progression [2, 3]. NF-*κ*B is also required to sustain a proinflammatory microenvironment that facilitates extracellular matrix (ECM) degradation and tumor cell dissemination in prostate cancer (PCa) [4, 5]. Clusterin (CLU) is a secreted glycoprotein known to be involved both in inflammation and cancer [6–8]. CLU may be retained inside the cell as a consequence of various stress-activated mechanisms, which include retrotranslocation from the endoplasmic reticulum, alternative splicing, alternative transcription initiation, and internalization from the extracellular compartment [9, 10]. Intracellular CLU interferes with relevant cell signaling pathways, including NF-*κ*B [11, 12]. *In vivo* CLU has anti-inflammatory functions; indeed, in the experimental model of induced autoimmune myocarditis and pancreatitis, CLU knockout mice (CLUKO) show signs of more severe inflammation and cellular pathology than CLU-expressing wild-type controls (WT) [13, 14]. CLU expression is altered in many tumors including PCa, although conflicting data about its tumor suppressive or tumor permissive role have been published [8]. We and other authors have observed that CLU is downregulated in human PCa progression [15, 16] and in tumors arising in the TRansgenic Adenocarcinoma of the Mouse Prostate (TRAMP) model [17, 18]. Moreover, CLUKO mice are more susceptible than WT to chemically induced skin tumorigenesis, suggesting that CLU might negatively modulate epithelial cell transformation [19]. When CLUKO mice were crossed with TRAMP to obtain TRAMP/CLUKO mice, we found that tumor spreading and metastases occurred earlier in animals lacking CLU expression [20]. Cancerous lesions of TRAMP prostates are positive for NF-*κ*B and Ki67 staining in comparison to WT. Of note, an even stronger expression of these proteins was detected in the cancerous lesions of age-matched TRAMP/CLUKO [20], suggesting the hypothesis that one possible mechanism by which CLU slows down tumor spreading in TRAMP mice might also involve limiting NF-*κ*B activity. The aim of the present work is to challenge the aforementioned hypothesis by an articulated experimental approach that started from examining the expression of p65 and CLU in large microarray dataset retrieved in GEO (Gene Expression Omnibus) which included expression profiling of normal, prostate primary tumors, and metastatic tissue. Then, we evaluated the changes of NF-*κ*B expression and activity in human PCa cells following either CLU overexpression or silencing. Finally, we measured in prostate tissue obtained from WT, CLUKO, TRAMP, and TRAMP/CLUKO, the expression and activity of ECM metalloproteinases (MMP-2 and MMP-9) that are known to be involved in tumor dissemination being regulated by the NF-*κ*B pathway.

## 2. Materials and Methods

### 2.1. Public Domain Data

mRNA expression levels of the human prostate sample from microarray dataset GSE6919 were analyzed with GEO2R web tool (http://www.ncbi.nlm.nih.gov/geo/geo2r/). GSE6919 was performed on the Affymetrix Human U95 Version 2 Array platform (GPL8300), which comprises the expression profiles of the following human specimens: 18 normal prostates, 63 normal prostates adjacent to the tumor, 65 primary prostate tumors, and 25 prostate metastases. Expression levels of CLU and p65 were compared between primary tumors and metastatic tissues. The Benjamini and Hochberg false discovery rate method was used to correct for multiple comparisons.

### 2.2. Cell Culture

PC3 cells were purchased from the American Tissue Culture Collection and were routinely grown in Ham's F12 : MEM medium (1 : 1). The culture media were supplemented with 10% foetal bovine serum (Lonza, Basel, CH), 2 mM L-glutamine, 100 U/mL penicillin, and 100 *μ*g/mL streptomycin. The cells were incubated at 37°C under a 5% CO_2_ atmosphere and harvested by trypsin/EDTA (Sigma-Aldrich, Steinheim, DE). The full-length human CLU coding sequence was previously cloned into the bicistronic expression vector pIREShyg1 (U89672, Clontech, Oxford, UK) [21]. PC3_CLU_ and PC3_mock_ were generated by clonal selection and maintained as previously described [21].

### 2.3. CLU Transient Overexpression

PC3 cells were seeded at a density of 7.5 × 10^4^ cells/mL and transfected with 4 *μ*g of pIRES-CLU or pIREShyg1 (mock) using FuGene® HD Transfection Reagent (Promega, Madison, WI). Cells were harvested 24 or 48 hours after transfection and used for mRNA and protein extraction.

### 2.4. CLU Silencing

PC3 cells were seeded at a density of 2 × 10^5^ cells/mL and transfected with 100 nM CLU siRNA (5′-GCAGCAGAGUCUUCAUCAU-3′ Ambion, Austin, TX) using the TransIT-TKO Transfection Reagent (Mirus Bio, Madison, WI). A universal scrambled sequence called NC siRNA (Integrated DNA Technologies, Coralville, IA) was used as negative control. Cells were harvested at 24 and 48 hours after transfection and used for RNA and protein extraction.

### 2.5. Genetically Modified Mice

Male and female TRAMP mice heterozygous for the SV40 transgene were obtained from Jackson Laboratories (Bar Harbor, ME, USA), maintained in a C57Bl/6 background and screened according to Greenberg et al. [22]. CLU-deficient mice (CLUKO) backcrossed to the C57Bl/6 strain for more than 10 generations were obtained breeding heterozygous parents and genotyping the offspring for CLU expression [13]. Female TRAMP mice were mated with homozygous (CLUKO) male mice to obtain TRAMP/CLU^+/−^ mice. Then, TRAMP/CLU^+/−^ females were mated with heterozygous (CLU^+/−^) male mice to obtain TRAMP/CLUKO mice. Mice were housed in a standard animal facility under controlled environmental conditions (22 ± 2°C, 12 hours light/dark cycle) and were allowed free access to food and water. For the immunohistochemical detection of p65, a total of 12 mice, 3 for each experimental group, were sacrificed at 12 and 24 weeks of age, the prostates were excised, fixed in 10% (vol/vol) neutral-buffered formalin at 4°C overnight, and processed to paraffin using standard procedures for immunohistochemical analysis. For the zymography assay, a total of 12 male mice, 3 for each experimental group (WT, CLUKO, TRAMP, and TRAMP/CLUKO) were euthanized at 36 weeks of age. Prostates were excised, snap-frozen in liquid nitrogen, and stored at −80°C until use. All the experimental procedures involving transgenic mice were approved and conducted in accordance with the Italian law (D.lgs 26/2014).

### 2.6. RNA Extraction, cDNA Preparation, and Real-Time RT-PCR (qPCR)

RNA was extracted from human cells or prostate samples using TRIzol® reagent (Fisher Molecular Biology, Rome, IT) and purified with the PureLink® RNA Mini Kit (Fisher Molecular Biology, Rome, IT). cDNA was prepared using the RevertAid First Strand cDNA Synthesis kit (Thermo Fisher Scientific, Waltham, MA) following the manufacturer's instructions [23]. Primers information is reported in Table 1. The human or murine glyceraldehyde 3-phosphate dehydrogenase (hGAPDH and mGAPDH) was used as the reference gene. Normalized CT (ΔCT) = CT (target gene) − CT (GAPDH) when the indicated ΔΔCT values were used for calculation of relative expression by the 2^−ΔΔCT^ method, where ΔΔCT = ΔCT target sample − ΔCT control sample.

### 2.7. Protein Extraction, SDS-PAGE, and Western Blot (WB) Analysis

Cells were lysed in the RIPA buffer (50 mM Tris-HCl pH 7.4, 100 mM NaCl, and 1% Triton X-100) supplemented with protease and phosphatase inhibitors (Sigma-Aldrich, Steinheim, DE). Protein concentration was determined using the Bio-Rad DC Protein Assay (Bio-Rad, Berkley, CA). 70 *μ*g of proteins/lane was separated on 12% SDS-PAGE gel, transferred to PVDF membranes, routinely stained with red Ponceau for loading and transfer control, and probed for 16 hours at 4°C by means of the following antibodies: mouse monoclonal anti-CLU, dilution 1 : 1000 (Millipore, Billerica, MA); rabbit monoclonal anti-p-p65_S536_, dilution 1 : 500, and mouse monoclonal anti-p65, dilution 1 : 1000 (Cell Signaling, Technology, Denver, MA); mouse monoclonal anti-*β*-actin, dilution 1 : 200 (Santa Cruz Biotechnology, Dallas, TX) and rabbit monoclonal anti-I*κ*B*α*, dilution 1 : 1000 (Cell Signaling Technology, Denver, MA); rabbit monoclonal anti-IKK*β*, dilution 1 : 1000 (Cell Signaling Technology, Denver, MA); and rabbit polyclonal anti-Akt, dilution 1 : 1000 (Cell Signaling Technology, Denver, MA). Membranes were incubated with suitable secondary antimouse or antirabbit IgG antibodies conjugated to horseradish peroxidase, dilution 1 : 5000 and 1 : 20000, respectively, (Sigma-Aldrich, Steinheim, DE) and developed by the Chemiluminescence Blotting Substrate, POD (Roche, Basel, CH). Quantification of band intensities was conducted using Quantity One® software (Bio-Rad, Berkley, CA). Intensity of each band was normalized for *β*-actin intensity from the same sample on the same blot.

### 2.8. Immunocytochemical Analysis

PC3_mock_ and PC3_CLU_ cells were cultured on a glass cover slip at a density of 0.75 × 10^5^ cell/mL for 72 hours, fixed and permeabilized with 1 : 1 methanol : acetone (for CLU detection), or fixed in 4% paraformaldehyde and permeabilized with methanol (for p-p65_S536_ detection). After blocking in horse serum 3% (vol/vol) in Dulbecco's modified phosphate-buffered saline (D-PBS), cells were incubated with a mouse monoclonal anti-CLU antibody, dilution 1 : 50 (Millipore, Billerica, MA) or with a rabbit polyclonal anti-p-p65, dilution 1 : 200 (Santa Cruz, Dallas, TX) in 3% BSA in D-PBS solution for 1 hour, conjugated with fluorescent anti-mouse Alexa Fluor 488 or anti‐rabbit Alexa Fluor 568 antibodies (Invitrogen, Carlsbad, CA), dilution 1 : 200 in 3% bovine serum albumin (BSA), stained with 4′,6-diamidino-2-phenylindole (DAPI) (Biotium Inc, Hayward, CA), and embedded in Mowiol (Sigma-Aldrich, Steinheim, DE). Fluorescence images were acquired with the 200 inverted fluorescence microscope equipped with an AxioCam HRc camera and the AxioVision 4.8 software (all from Carl Zeiss, Göttingen, DE).

### 2.9. Cell Proliferation Assay

Cell proliferation of PC3_mock_ and PC3_CLU_ cells was evaluated by crystal violet assay. Briefly, cells were seeded at a density of 7.5 × 10^4^ cell/mL. At 24–48–72–96 hours, the medium was removed and the cells were fixed with 4% paraformaldehyde for 20 min at room temperature. Fixed cells were stained with 0.5% crystal violet (Sigma-Aldrich, Steinheim, DE) in 20% methanol for 15 min and then washed and dissolved in a solution of 0.1 M sodium citrate in ethanol 50%, pH 4.2. The absorbance at 540 nm was measured using a biophotometer (Eppendorf, Hamburg, DE).

### 2.10. Cell Cycle Analysis

PC3_mock_ and PC3_CLU_ cells were collected, washed in 1% BSA in PBS, fixed with 70% cold ethanol, and stored at −20°C overnight. DNA staining was performed following resuspension of the pellet in the buffer containing 20 *μ*g/mL propidium iodide (Invitrogen, Milan, Italy) and 0.1 mg/mL RNase (Sigma-Aldrich, Steinheim, DE) in PBS. The cell suspension was then analyzed for DNA content and cell cycle phase distribution by FACS analysis (Becton Dickinson, Franklin Lakes, NJ). The data were analyzed using the CellFIT 3.0.1 software (Becton Dickinson, Franklin Lakes, NJ).

### 2.11. NF-*κ*B Activity Luciferase Assay

12.5 × 10^4^ cells/mL were seeded and transfected with 0.2 *μ*g of pNF*κ*B-LUC reporter plasmid (631904, Clontech, Mountain View, CA) or cotransfected with 0.1 *μ*g of pNF*κ*B-LUC and 0.1 *μ*g of pIRES-CLU or pIREShyg1 or 1 pmol of CLU siRNA or NC siRNA using Lipofectamine® 3000 (Thermo Fisher Scientific, Waltham, MA). The luciferase activity was measured 24 hours after transfection using the Britelite™ plus reactive (PerkinElmer, Waltham, MA) using the EnSpire® Multimode Plate Readers (PerkinElmer, Waltham, MA) and normalized to the total protein content after checking for equal transfection efficiency. The results are representative of three independent experiments run in triplicate.

### 2.12. Immunohistochemical Analysis

Paraffin-embedded tissue sections (5 mm) mounted onto glass slides were hydrated in xylene, graded alcohol. Antigen retrieval was performed by microwave (5 min at 700 W) using a sodium citrate buffer (10 mM, pH 6.0). Endogenous peroxidase activity was quenched with 3% H_2_O_2_. Nonspecific binding was blocked with swine serum diluted 1 : 10 in BSA 1%. Immunostaining was performed using the mouse monoclonal anti-p65 antibody dilution 1 : 50 in BSA 1% (Santa Cruz Biotechnology, Dallas, TX) and incubated for 1 h at room temperature. From the secondary antibody to the chromogen reaction, a Universal LSAb2 HRP kit (DakoCytomation, Glostrup, DK) was used according to the manufacturer's instruction as previously described [20].

### 2.13. Gelatin Zymography

Frozen mouse prostates were finely grinded with liquid nitrogen and lysed in a lysis buffer (25 mM Tris-HCl pH 7.4, 100 mM NaCl, 1% IGEPAL® CA-630, Sigma-Aldrich, Steinheim, DE) supplemented with 1 : 100 protease and phosphatase inhibitors (Sigma-Aldrich, Steinheim, DE). Protein concentration was determined using the Bio-Rad DC Protein assay (Bio-Rad, Berkley, CA). 30 *μ*g of proteins/lane was separated on 8% SDS-PAGE gel containing 0.1% porcine skin gelatine (Sigma-Aldrich, Steinheim, DE). Gels were washed for 30 min at room temperature in a washing buffer (2.5% Triton X-100) and incubated for 18 hours at 37°C in an activation buffer (20 mM Tris-HCl pH 8.0, 5 mM CaCl_2_, and 150 mM NaCl). Gels were stained with 0.1% Coomassie Brilliant Blue R250 (Sigma-Aldrich, Steinheim, DE) and destained with a destain solution (10% ethanol and 10% acetic acid).

### 2.14. Immunoprecipitation Assay

500 *μ*g of intracellular protein from PC3_mock_ and PC3_CLU_ cells was precleared with 50 *μ*L of protein G-agarose beads (Roche, Basel, CH) for 30 min at 4°C with gentle rotation. Then, the lysates were incubated for 18 hours at 4°C with 5 *μ*g of the following antibodies: mouse monoclonal anti-CLU (05-354, Millipore, Billerica, MA), mouse monoclonal anti-p65 (#6956, Cell Signaling Technology, Denver, MA), and normal mouse IgG (NI03, Millipore, Billerica, MA). 50 *μ*L of protein G-Agarose beads (Roche, Basel, CH) was added and incubated for 4 hours at 4°C with gentle rotation. After centrifugation, immune-precipitated pellets were washed four times with ice-cold RIPA buffer, dissolved in the Laemmli buffer added with 100 nM dithiothreitol, and heated at 100°C for 3 min for reverse cross-link.

### 2.15. Statistical Analyses

The IBM SPSS statistical package (International Business Machines Corporation, Armonk, NY, USA, version 24) was used. Normal distribution of variables was checked by means of the Kolmogorov–Smirnov test. Statistics of variables included mean ± standard deviation (SD), the unpaired Student's *t*-test (luciferase assay and qPCR), and the Mann–Whitney *U* test (qPCR data). Statistical significance was set at *p* < 0.05. Pearson's correlation test on microarray data (*n*=80) was performed with GraphPad Prism 6 software. Details on the specific test used are reported in the figure legend of each experiment.

## 3. Results

### 3.1. CLU and p65 Are Significantly Inversely Correlated in Human Samples of PCa Metastases

**Figure 1:**
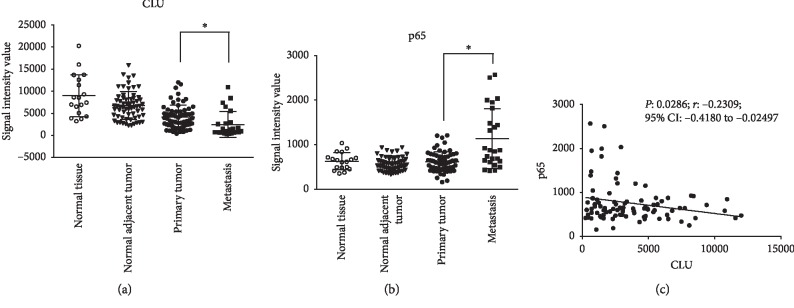
Expression of CLU and p65 mRNA in human prostate tissue samples. (a) mRNA expression of CLU and (b) p65 in human normal prostate tissue (○), normal prostate tissue adjacent to the tumor (▼), primary tumor (•), and metastasis (■) downloaded from GSE6919 dataset. The GEO2R tool was used to test differences in the mRNA levels between primary tumor and metastasis. Horizontal lines show the mean ± SD. ^*∗*^*p* < 0.0001 (*t*-test corrected for multiple comparison by the Benjamini and Hochberg method). (c) Dot plot visualization of the inverse correlation between the expression of CLU and p65 in human tumor samples (primary tumors and metastasis) from the GSE6919 dataset. Correlation coefficient was evaluated by Pearson's correlation test (*n*=80).

The GEO2R web tool was used to compare the mRNA expression of CLU and p65 in the microarray dataset GSE6919, which comprises primary and metastatic prostate tumors, normal prostate tissue adjacent to tumor, and normal prostate tissue from healthy donors (Figure 1(a)). The highest expression of CLU was found in normal prostate tissue and gradually decreased in normal tissue adjacent to the tumor and primary prostate tumor, reaching the lowest value in metastatic samples. A statistically significant decrease in CLU along with a significant increase in p65 expression was observed when tissues from primary and metastatic PCa were compared (Figure 1(a)). Moreover, in primary tumor and metastases, the expression of CLU and p65 is inversely correlated (Figure 1(b)).

### 3.2. NF-*κ*B Transcriptional Activity Is Inhibited by CLU Overexpression

**Figure 2:**
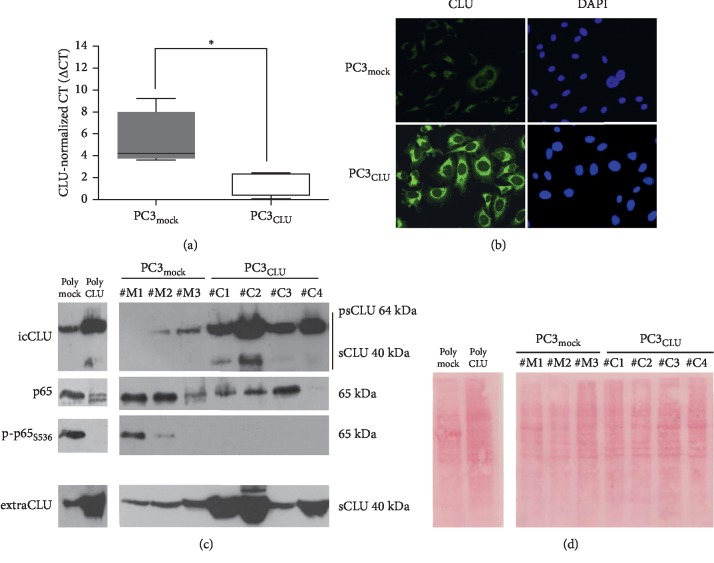
CLU stable overexpression and p65 expression and phosphorylation in PC3 cells. (a) Quantification of CLU mRNA in PC3_mock_ (namely, clones #M1, #M2, and #M3) and PC3_CLU_ (namely, clones #C1, #C2, #C3, and #C4) by qPCR. ΔCT values have been reported in a box plot graph; the line crossing the boxes represents the median value of the distribution. hGAPDH was used as a housekeeper gene. ^*∗*^*p* < 0.05 vs. PC3_mock_ (the unpaired Student's *t*-test). (b) Subcellular localization of CLU (green fluorescence) in PC3_mock_ and PC3_CLU_ cells. Nuclei staining with DAPI (blue fluorescence). The pictures shown refer to #M2 and #C2 clones and are representative of all the other analyzed clones. Image magnification was 32×. (c) Quantification of intracellular CLU, p65, and p-p65_S536_ proteins by WB analysis in PC3_mock_ (namely, clones #M1, #M2, and #M3), PC3_CLU_ (namely, clones #C1, #C2, #C3, and #C4), and polyclonal samples. (d) Red Ponceau staining was used as loading control. Quantification of CLU protein secreted in cell culture media by WB analysis in PC3_mock_ (namely, clones #M1, #M2, and #M3), PC3_CLU_ (namely, clones #C1, #C2, #C3, and #C4), and polyclonal samples. icCLU, intracellular CLU; extraCLU, CLU secreted in culture media; psCLU, uncleaved CLU precursor, 64 kDa; sCLU, cleaved mature CLU, 40 kDa.

**Figure 3:**
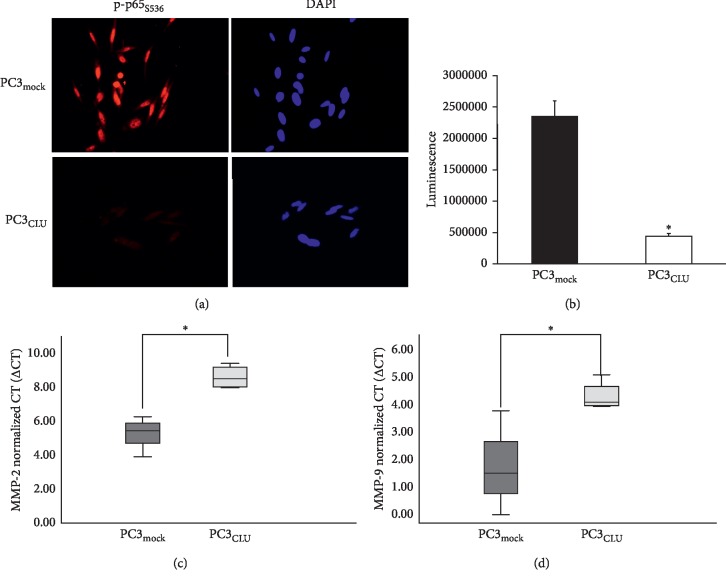
Effects of CLU stable overexpression on NF-*κ*B activation. (a) Subcellular localization of p-p65_S536_ (red fluorescence) in PC3_mock_ and PC3_CLU_ cells. Nuclei staining with DAPI (blue fluorescence). Image magnification was 32×. The pictures shown refer to #M2 and #C2 clones and are representative of all the other analyzed clones. (b) Luciferase assay for NF-*κ*B activity was carried out in PC3_mock_ (#M2) and PC3_CLU_ (#C2) cells transfected with the reporter vector pNF*κ*B-LUC. The assay was performed 24 hours after transfection. Mean normalized data of luminescence (arbitrary units) ± SD from six replicate wells of three independent experiments are reported on the *Y* axis. ^*∗*^*p* < 0.001 vs. PC3_mock_ (unpaired Student's *t*-test). (c) Quantification of MMP-2 and MMP-9 mRNA in PC3_mock_ and PC3_CLU_ clones by qPCR. ΔCT values are reported on the *Y* axis. hGAPDH was used as the housekeeper gene. ^*∗*^*p* < 0.05 vs. PC3_mock_ (the Mann–Whitney *U* test).

**Figure 4:**
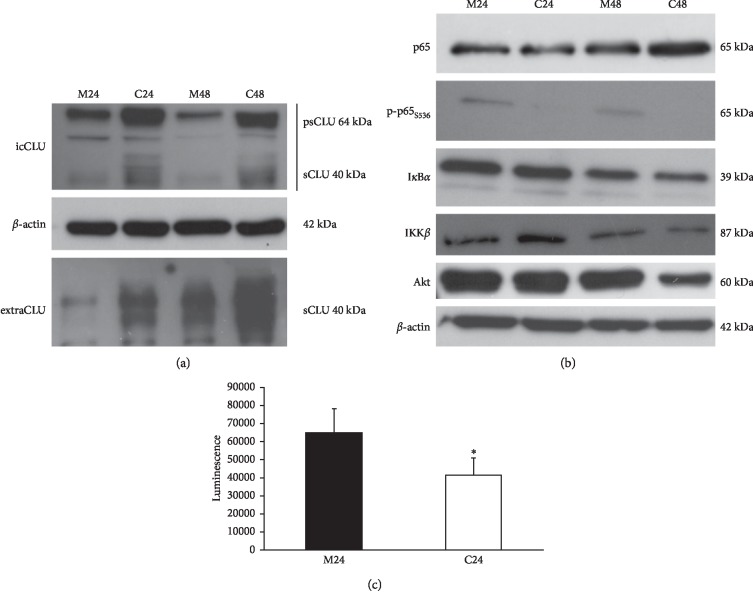
Effects of CLU transient overexpression on NF-*κ*B activation. Quantification of CLU (a) and p65, p-p65_S536_, I*κ*B*α*, IKK*β*, and Akt (b) protein by WB analysis in PC3 cells transfected with pIRES-mock (M) or pIRES-CLU (C). The analysis was carried out in whole cell lysates, and cell culture media was collected 24 and 48 hours after transfection. *β*-Actin was used as loading control. icCLU, intracellular CLU; extraCLU, CLU secreted in culture media; psCLU, uncleaved CLU precursor, 64 kDa; sCLU, cleaved mature CLU, 40 kDa. The data shown are representative of three independent experiments. (c) Luciferase assay for NF-*κ*B activity in PC3 cells cotransfected with pNF*κ*B-LUC plasmid and pIRES-mock (M24) or pIRES-CLU (C24). The assay was performed 24 hours after transfection. Mean normalized data of luminescence (arbitrary units) ± SD from six replicate wells of three independent experiments are reported on the *Y* axis. ^*∗*^*p* < 0.05 vs. M24 (the unpaired Student's *t*-test).

At least five CLU (PC3_CLU_) and five mock clones (PC3_mock_) were analyzed for CLU content, cell morphology, cell proliferation, and percentage of distribution of cells in different phases of the cell cycle. CLU mRNA is significantly more expressed in PC3_CLU_ than in PC3_mock_ (Figure 2(a)). Both the precursor form of 64 kDa (psCLU) and the mature form of 40 kDa (sCLU) were expressed at a higher level in cell lysates and culture media of PC3_CLU_ compared to PC3_mock_, although the intensity of the WB blot bands shows some differences depending on the single clone analyzed ([Supplementary-material supplementary-material-1]). These results have also been confirmed in a polyclonal population obtained by pooling together several PC3_CLU_ clones different from those analyzed individually (Figure 2(c)). The immunofluorescence analyses confirmed that CLU is significantly more expressed in PC3_CLU_ than in PC3_mock_ and localizes to the cytoplasm (Figure 2(b)). PC3_CLU_ cells are more round shaped than PC3_mock_ ([Supplementary-material supplementary-material-1]) and grow slower than PC3_mock_ ([Supplementary-material supplementary-material-1]). We also measured an impairment in the cell cycle distribution, which is consistent with a slowdown of the progression between the G0/G1 and the S phase in PC3_CLU_ compared to PC3_mock_ ([Supplementary-material supplementary-material-1]). When, in the same clones, we measured the expression of NF-*κ*B (p65 total) and active NF-*κ*B (p-p65_S536_), we detected a reduction of p-p65_S536_ in PC3_CLU_ compared to controls ([Supplementary-material supplementary-material-1]), also in the polyclonal samples that ideally represent the averaged trend of many cell clones (Figure 2(c)). The immunocytochemical (IC) analysis showed that p-p65_S536_ is reduced in PC3_CLU_ compared to PC3_mock_, where, in the contrary, an intense staining was found in the cell nuclei (Figure 3(a)). The transcriptional activity of NF-*κ*B measured by the luciferase assay is reduced in PC3_CLU_ compared to PC3_mock_ (Figure 3(b)). Consistently, the expression of ECM-degrading enzymes, such as MMP-2 and MMP-9, whose expression is transcriptionally controlled by NF-*κ*B, is significantly lower in PC3_CLU_ clones compared to PC3_mock_ (Figure 3(c)). Similarly, CLU expression (psCLU and sCLU) was increased in cell lysates and in growth media of PC3 cells transiently transduced with p-IRES-CLU for 24 and 48 hours (Figure 4(a), lanes C24 and C48) in comparison to cells receiving the empty vector (Figure 4(a), lanes M24 and M48). In the same cells, p-p65_S536_ is significantly reduced (Figure 4(b), lanes C24 and C48) compared to mock (Figure 4(b), lanes M24 and M48) and is accompanied by a reduction of the transcriptional activity of NF-*κ*B, measured by the luciferase assay (Figure 4(c)). A slight reduction in I*κ*B*α* expression along with a significant decrease in IKK*β* and Akt was detected in C48 in comparison to M48 (Figure 4(b)).

### 3.3. CLU Silencing Increases NF-*κ*B Transcriptional Activity

**Figure 5:**
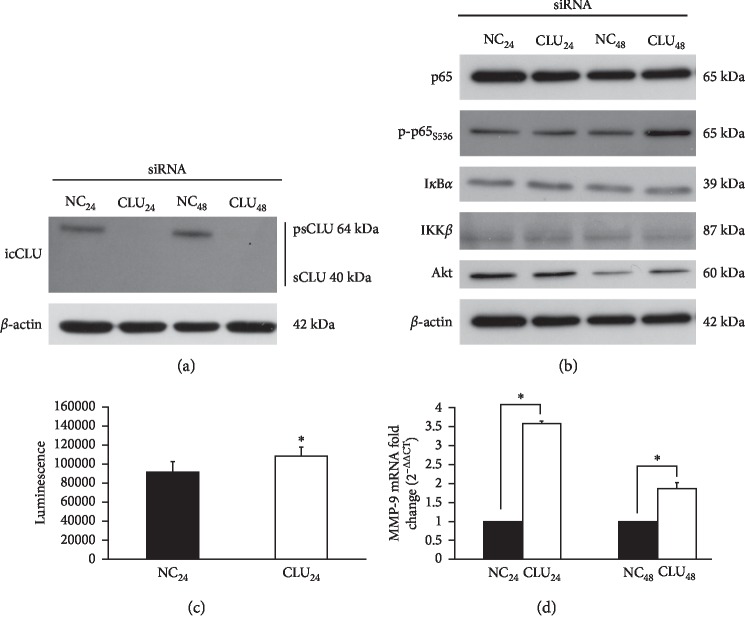
Effects of CLU silencing on p65 phosphorylation and NF-*κ*B activation. Quantification of CLU (a) or p65, p-p65_S536_, I*κ*B*α*, IKK*β*, and Akt (b) protein by WB analysis in PC3 cells transfected with siRNA-CLU (CLU) or siRNA negative control (NC) in whole cell lysates 24 and 48 hours after transfection. *β*-Actin was used as loading control. icCLU, intracellular CLU; psCLU, uncleaved CLU precursor, 64 kDa; sCLU, cleaved mature CLU, 40 kDa. The data shown are representative of three independent experiments. (c) The luciferase assay for NF-*κ*B activity in PC3 cells cotransfected with pNF*κ*B-LUC plasmid and siRNA-CLU or NC. The assay was performed 24 hours after transfection. Mean normalized data of luminescence (arbitrary units) ± SD from six replicate wells of three independent experiments are reported on the *Y* axis. ^*∗*^*p* < 0.05 vs. M24 (the unpaired Student's *t*-test). (d) Quantification of MMP-9 mRNA by qPCR in PC3 cells transfected with siRNA-CLU or NC for 24 or 48 hours. 2^−ΔΔCT^ values are reported on the *Y* axis. GAPDH was used as the housekeeper gene. The value of MMP-9 expression in NC samples was fixed equal to 1. Error bars represent SD of three independent determinations each performed in duplicate. ^*∗*^*p* < 0.01 vs. NC (the unpaired Student's *t*-test).

CLU expression was almost completely abrogated in PC3 cells, starting from 24 and up to 48 hours after CLU siRNA transfection in comparison to negative control (NC) transduced cells (Figure 5(a)). In the same samples, p-p65_S536_ increased after 24 hours and much more significantly after 48 hours in CLU siRNA (Figure 5(b), lanes CLU 24 and CLU 48) compared to NC transduced cells (Figure 5(b) lanes NC 24 and NC 48). I*κ*B*α* and IKK*β* did not change, while a significant increase of Akt was detected in CLU 48 in comparison to NC 48 (Figure 5(b)). By the Luciferase assay, we found that NF-*κ*B transcriptional activity increased in PC3 cells transduced with CLU siRNA in comparison to NC (Figure 5(c)). Consistently, the expression of MMP-9 mRNA is significantly increased 24 and 48 hours after CLU siRNA transfection in comparison to NC (Figure 5(d)).

### 3.4. CLU Does Not Prevent NF-*κ*B Activation by means of a Direct Interaction with the p65 Subunit

**Figure 6:**
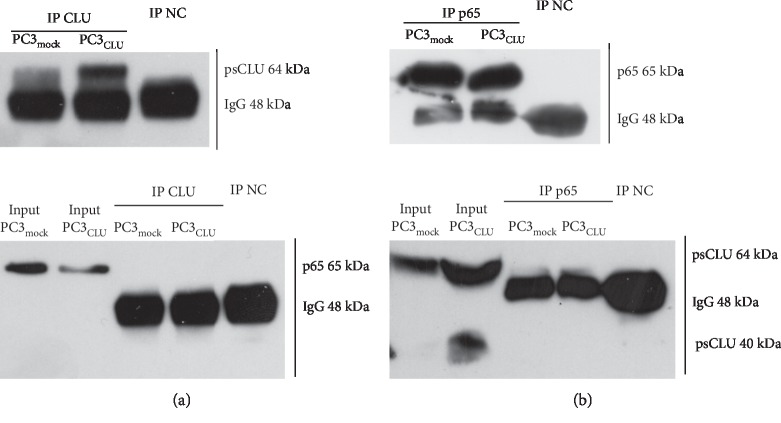
CLU and p65 interaction in PC3_mock_ and PC3_CLU_ cells. Total proteins from PC3_mock_ and PC3_CLU_ cells were immunoprecipitated with anti-CLU (IP CLU) (a) or anti-p65 (IP p65) (b) followed by WB with anti-p65 and anti-CLU antibodies. In parallel, immunoprecipitation with IgG was performed as negative control (IP NC). The specificity (negative control) and effectiveness (positive control) of the immunoprecipitation are shown in the upper panel of a and b. The interaction between CLU and p65 was evaluated comparing the amount of p65 recovered in the fraction immunoprecipitated with CLU (IP CLU) to the amount of p65 detected in the input whole cell lysate ((a), lower panel) or vice versa comparing the amount of CLU recovered in the fraction immunoprecipitated with p65 to the amount of CLU detected in the input whole cell lysate ((b), lower panel).

We investigated whether CLU may physically hide the accessibility of S_536_ to specific kinases such as IKK*β* by direct binding with p65. Therefore, we immune-precipitated (IP) CLU and p65 from PC3_CLU_ and PC3_mock_ cell lysates. Then, we searched for CLU and p65 physical interaction by WB analysis of the IP fractions. CLU was successfully pulled down when the specific anti-CLU antibody was used for immunoprecipitation (IP positive control), as demonstrated by the presence of a band at 64 kDa in the IP fraction (Figure 6(a), upper panel). The result of the immunoprecipitation reaction is specific because no CLU band is detectable in the mouse IgG immunoprecipitated sample (negative control). No bands were detected, instead, when the same membrane was probed with an anti-p65 antibody, indicating that no direct interaction took place between CLU and p65 in PC3_CLU_ compared to PC3_mock_ (Figure 6(a), lower panel). Similarly, when the intracellular lysates were immunoprecipitated with an anti-p65 antibody, we were able to detect p65 in the IP fraction (positive control), while no p65 was detected in the mouse IgG immunoprecipitated sample (negative control) (Figure 6(b), upper panel). No bands were detected, instead, when the same membrane was probed with an anti-CLU antibody (Figure 6(b), lower panel).

### 3.5. MMP-2 and MMP-9 Gelatinolitic Activity Increased in TRAMP/CLUKO Mice Compared to TRAMP Littermates

**Figure 7:**
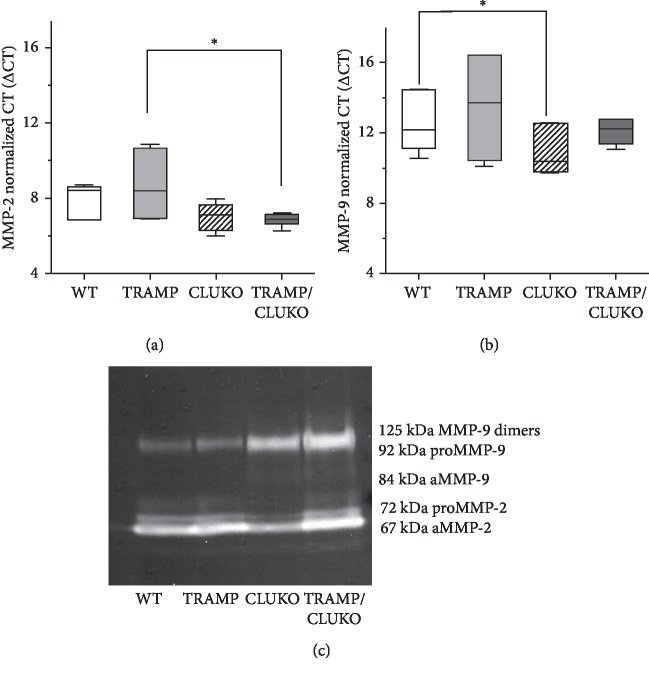
Effects of CLU knockout on MMP-2 and MMP-9 expression and activity *in vivo*. Quantification of MMP-2 (a) and MMP-9 (b) mRNA in prostate samples of WT, TRAMP, CLUKO, and TRAMP/CLUKO mice. Number of animals in each group = 3. ΔCT values are reported on the *Y* axis. The m-GAPDH was used as teh housekeeper gene. ^*∗*^*p* < 0.05 (the Mann–Whitney *U* test). (c) Gelatin zymography for MMP-2 and MMP-9 in prostate samples of WT, TRAMP, CLUKO, and TRAMP/CLUKO mice. The image shown is representative of three independent experiments. proMMP-9, uncleaved MMP-9; aMMP-9, cleaved active MMP-9; proMMP-2, uncleaved MMP-2; aMMP-2, cleaved active MMP-2.

By immunohistochemical analysis, we observed that p65 is more expressed in prostate tissues of TRAMP/CLUKO mice than age-matched TRAMP animals expressing CLU ([Supplementary-material supplementary-material-1]). Then, we proceeded measuring the expression level and the enzymatic activity of MMP-2 and MMP-9 in prostate samples obtained from WT, CLUKO, TRAMP, and TRAMP/CLUKO littermates. The lowest median values of normalized CT for MMP-2 were measured in the CLUKO and TRAMP/CLUKO prostates by qPCR analysis of mRNA expression. The increase in MMP-2 expression is statistically significant when TRAMP/CLUKO is compared with TRAMP (Figure 7(a)). The lowest median values of normalized CT for MMP-9 were measured in CLUKO mice, in which MMP-9 expression is therefore significantly upregulated compared to WT littermates (Figure 7(b)). The enzymatic activity of MMP-2 and MMP-9 was assessed by gelatine zymography. A strong increase in MMP-9 and MMP-2 gelatinolitic activity was observed in both CLUKO and TRAMP/CLUKO mice in comparison to WT and TRAMP mice, respectively. The strongest difference was observed for the pro-MMP-9 band, corresponding to the uncleaved precursor protein. Additional bands at lower molecular weight, corresponding to the cleaved active enzymes (aMMP-9 and a-MMP-2), are more expressed in the prostate tissues of animals that lack CLU expression.

## 4. Discussion

Although the adaptive immune system mediates antitumor effects through immune surveillance, many tumors acquire the ability to subvert inflammatory signals to their benefit [4]. NF-*κ*B is aberrantly activated in the majority of PCa cases, and it is considered the major transcription factor contributing to sustain a proinflammatory, tumor-permissive microenvironment [24]. Although many molecules act as NF-*κ*B activators, very few proteins are known to negatively interfere with NF-*κ*B signaling, a part from I*κ*Bs proteins, which are the major inhibitors of the pathway. CLU is a heavy glycosylated secreted heterodimeric protein that is involved in many processes characterized by cellular stress, like ageing, chronic tissue inflammation, metabolic diseases, and cancer [8, 25]. CLU mRNA is downregulated in the vast majority of naïve cancers according to Oncomine® database, and PCa does not make exception [6]. The analyses performed on the microarray dataset GSE6919 with GEO2R web tool showed that CLU expression is reduced in PCa in comparison to normal prostate tissue, following an ideal decreasing gradient from normal healthy prostate to epithelial tissues adjacent to primary tumors and then primary tumors up to metastases. Interestingly, the expression of NF-*κ*B is significantly increased in metastases, where it inversely correlates with CLU expression. On the one hand, these results support early reports from our group [15, 16, 26] and agree with the finding that histone tails modification, including H3 pan-deacetylation and H3K27 trimethylation as well as CpG island hypermethylation at the CLU promoter contribute to the epigenetic repression of this gene both in human PCa cell lines and in the TRAMP model [17, 27]. On the other hand, the same results highlight that the inverse relation between CLU and NF-*κ*B expression, previously noticed in CLUKO and TRAMP/CLUKO mice [20], can be extended from the animal model to the human disease.

Previously, some *in vitro* results have suggested that the relation between NF-*κ*B and CLU is complex, in that CLU is transcriptionally upregulated by NF-*κ*B [28] and, at the same time, intracellular CLU can inhibit NF-*κ*B activation by stabilizing the inhibitor I*κ*B [7, 11, 12, 29]. In addition to the I*κ*B-mediated regulation of NF-*κ*B nuclear translocation, several studies have shown that the NF-*κ*B proteins are post-translationally modified. These changes may directly control not only dimer interaction with other factors, their stability, and turnover but also may contribute to the selective regulation of NF-*κ*B transcriptional activity in a gene-specific manner. Altogether, these events contribute to a multifaceted NF-*κ*B signaling control that is enormously more sophisticated than a simple on/off switch [30]. By gain-of-function and loss-of-function experiments in androgen-independent PCa cell line PC3, we demonstrated that CLU expression is required to reduce the amount of constitutively active NF-*κ*B, by decreasing p-p65_S536_, without significantly affecting I*κ*B*α* expression. Indeed, keeping in mind that I*κ*B*α* is a direct transcriptional target of NF-*κ*B, the slight reduction of I*κ*B*α* measured in PC3 cells overexpressing CLU 48 hours after transfection is a direct consequence of the inhibition of NF-*κ*B transcriptional activity measured in these cells [31, 32].

Indeed, following ectopic expression of CLU c-DNA, the expression of p-p65_S536_ is reduced along with p65 fraction translocated in the cell nuclei in comparison to empty vector transfected cells. The S_536_ phosphorylation site is located in the transactivation domain of p65 and is conserved in both human and mouse [33]. It has been observed that the constitutive phosphorylation of p65 at serine 536 (p-p65_S536_) affects the p65 dimer composition as well as the selective recruitment of phospho-p65 to specific promoters [34]. Of note, p-p65_S536_ is increased in patients carrying TMPrSS2/ERG (T/E) fusion, the most common gene rearrangement in PCa, where it is suggested to play a critical role in PCa tumorigenesis by enhancing the p65 transcriptional activity of both proinflammatory chemokines and ECM-modifying enzymes, thus producing a tumor-permissive microenvironment [35]. Under this experimental condition, the luciferase assay confirms a reduction of NF-*κ*B transcriptional activity. The expression of NF-*κ*B transcribed genes involved in ECM degradation, such as MMP-9 and MMP-2, is consistently reduced. In order to exclude any possible bias coming from the unphysiological condition of protein overexpression, we integrated our experimental design with a complementary approach of gene function modulation by silencing CLU through siRNA oligonucleotides. After silencing CLU in PC3 cells, we observed opposite results compared to those detected in CLU overexpression models, i.e., an increase of p-p65_S536_ and an increase of Nf-*κ*B activity accompanied by an increase of MMP-9 expression. Our results are in good agreement with previous findings from other authors in different models [11, 12, 29]. A unique study showed that NF-*κ*B is activated by CLU-mediated stabilization of COMMD1, a protein that facilitates I*κ*B ubiquitination and proteasomal degradation [36]. We observed that CLU regulates NF-*κ*B activity by reducing p65 phosphorylation at S_536_. Because p-p65_S536_ does not bind I*κ*B*α* nor is proteasome degraded, we suggest that CLU might inhibit NF-*κ*B constitutive activation by an I*κ*B-independent pathway [34]. The mechanism is supposed to be particularly relevant in androgen-independent cells such as PC3 and DU145 that constitutively express high levels of active Nf-*κ*B, being almost unresponsive to TNF*α* stimulation [37]. Based on the results of the IP analysis, we excluded a direct binding between CLU and p65 and suggest that CLU regulates the amount of intracellular p-p65_S536_ by regulating the activity of upstream kinases as IKK*β* or Akt by a mechanism similar to that observed in cancer cells under growth-constraining condition [38]. This hypothesis is corroborated by the fact that Akt and IKK*β* expression are reduced in PC3 cells overexpressing CLU for 48 hours in comparison to mock transfected controls. On the contrary, Akt expression is increased in PC3 cells silenced for CLU for 48 hours compared to NC-transduced control cells. Although the present study was conducted in a single cell line, which is in fact a limitation of the experimental design, we previously showed that CLU stable overexpression in prostate epithelial immortalized cells (PNT1a) inhibits NF-*κ*B activity by reducing Akt expression [20].

The finding that CLU reduces MMP-9 and MMP-2 expression by inhibiting NF-*κ*B is biologically relevant for PCa because these enzymes play a role in tumor dissemination by degrading ECM and basement membrane. Conflicting data have been reported about CLU effects on MMP expression in cultured cells. Administration of highly supraphysiological amounts of exogenous purified CLU in a murine macrophage cell line increased MMP-9 expression and activity. Disappointingly, no effects were observed following CLU silencing, questioning the specificity of the observed phenomena [39]. CLU increased MMP-2 expression in hepatocellular carcinoma cells [40], while CLU overexpression inhibited MMP-9 expression by reducing NF-*κ*B activation in vascular smooth muscle cells [41]. CLU prevented stress-induced MMP-9 aggregation and activation of various MMP, both inside and outside human epithelial cells, by direct binding with the catalytic MMP domain [42]. To the best of our knowledge, our study is the first one to report the effects of CLU abrogation on MMP expression and activity *in vivo*, in a preclinical model of PCa. We found that MMP-2 and MMP-9 expression is higher in prostate samples from CLUKO and TRAMP/CLUKO mice in comparison to WT and TRAMP littermates. Moreover, the results of zymography assay show that MMP-2 and MMP-9 activity is higher in the prostate of CLU-null mice. These data are consistent with the observation that tumors in TRAMP/CLUKO mice showed increased tendency to metastasize when comparing with tumors in TRAMP mice expressing normal levels of CLU [20].

## 5. Conclusions

Overall, the presented data support the hypothesis that in normal physiological condition CLU participates to a negative loop in which transcriptional activation of CLU by NF-*κ*B is evoked to dampen the excessive constitutive activation of NF-*κ*B itself. When CLU expression is reduced in PCa by epigenetic mechanisms, the physiological brake on NF-*κ*B might be relieved. This effect is more important in the late PCa stage, when Nf-*κ*B activity and expression reach the maximum. In fact, the increased NF-*κ*B activity may cause an increased expression of proteases involved in ECM degradation that favors PCa progression towards a metastatic disease. Considering the important involvement of inflammatory processes in PCa onset and progression, the pharmacological strategies aimed at counteract epigenetic CLU downregulation in early stages of PCa could be crucial for effectively controlling the signaling pathways that lead to proliferation and invasion in advanced cancers.

## Figures and Tables

**Table 1:** Sequences of the primers used in qPCR.

Gene	Primer for 5′ ⟶ 3′	Primer rev 5′ ⟶ 3′	*T *_a_ (°C)	Cycle
hCLU	TGATCCCATCACTGTGACGG	GCTTTTTGCGGTATTCCTGC	60	40
hMMP-2	AGCGCTACGATGGAGGCGCTA	AGAAGGTGTTCAGGTATTGCACTGC	60	40
hMMP-9	GCGCCAGCGAGGTGGACCGGA	ACGGGAGCCCTAGTCCTCAGGGCAC	65	40
hGAPDH	AACCTGCCAAATATGATGAC	TTGAAGTCAGAGGAGACCAC	60	40
mMMP-2	GGTGGTGGTCATAGCTACTTC	TGAAGATGATAGGGCCCGTG	60	40
mMMP-9	TTGAGTCCGGCAGACAATCC	ACTTCCAGTACCAACCGTCC	60	40
mGAPDH	TCAAGCTCATTTCCTGGTAT	GTCCAGGGTTTCTTACTCCT	60	40

## Data Availability

No data were used to support this study.

## Supplementary Materials

Supplementary Materials Figure S1: p65 expression and phosphorylation in additional PC3mock, PC3CLU, and p-p65S536 clones. Quantification of CLU, p65, and p-p65S536 protein by WB analysis in PC3CLU (namely, clones #C5, #C6, and #C7) and PC3mock (namely, clone #M4). Red Ponceau staining was used for loading and transfer control. icCLU, intracellular CLU; psCLU, uncleaved CLU precursor, 64 kDa; sCLU, cleaved mature CLU, 40 kDa. Figure S2: morphology, proliferation, and cell cycle analysis of PC3mock and PC3CLU clones. (a) Phase-contrast images of PC3 cells, PC3mock, and PC3CLU. Magnification: 20×. (b) Cellular proliferation was assessed in PC3mock (black square) and in PC3CLU (white square) clones at the indicated time after seeding by crystal violet assay. Error bars represent SD of the mean of three PC3mock and three PC3CLU clones. ^*∗*^*p* < 0.05 (the unpaired *t*-test vs. mock). (c) Cell cycle analysis was carried out in PC3mock and PC3CLU by FACS analysis. The graphs are representative of all the clones analyzed. The percentage of distribution of the cells in the cell cycle phases is reported in the table. Figure S3: expression of p65 in TRAMP and TRAMP/CLUKO prostate tissues. Immunohistochemical staining of p65 in prostate tissues of 12- and 24-week-old (left and right panels, respectively) TRAMP and TRAMP/CLUKO mice. Three animals for each experimental group were examined. Images magnification 40×.
