# Correction to “Clusterin Silencing in Prostate Cancer Induces Matrix Metalloproteinases by an NF‐κB‐Dependent Mechanism”

**DOI:** 10.1155/jo/9894523

**Published:** 2026-05-31

M. Bonacini, A. Negri, P. Davalli, et al., “Clusterin Silencing in Prostate Cancer Induces Matrix Metalloproteinases by an NF‐κB‐Dependent Mechanism,” *Journal of Oncology* 2019, no. 1 (2019): 4081624, https://doi.org/10.1155/2019/4081624.

In the article titled, “Clusterin Silencing in Prostate Cancer Induces Matrix Metalloproteinases by an NF‐κB‐Dependent Mechanism,” there was an error in Figure 5b. Specifically, the bands representing the expression of p65 were erroneously selected to also represent the expression of β‐actin. This error was made during the figure preparation process. The correct Figure 5 is as follows:

FIGURE 5Effects of CLU silencing on p65 phosphorylation and NF‐κB activation. Quantification of CLU (a) or p65, p‐p65S536, IκBα, IKKβ, and Akt (b) protein by WB analysis in PC3 cells transfected with siRNA‐CLU (CLU) or siRNA negative control (NC) in whole cell lysates 24 and 48 hours after transfection. β‐actin was used as loading control. icCLU, intracellular CLU; psCLU, uncleaved CLU precursor, 64 kDa; sCLU, cleaved mature CLU, 40 kDa. The data shown are representative of three independent experiments. (c) The luciferase assay for NF‐κB activity in PC3 cells cotransfected with pNFκB‐LUC plasmid and siRNA‐CLU or NC. The assay was performed 24 hours after transfection. Mean normalized data of luminescence (arbitrary units) ± SD from six replicate wells of three independent experiments are reported on the *Y* axis. ^∗^ *p* < 0.05 vs. M24 (the unpaired Student′s *t*‐test). (d) Quantification of MMP‐9 mRNA by qPCR in PC3 cells transfected with siRNA‐CLU or NC for 24 or 48 hours. 2^−ΔΔCT^ values are reported on the *Y* axis. GAPDH was used as the housekeeper gene. The value of MMP‐9 expression in NC samples was fixed equal to 1. Error bars represent SD of three independent determinations each performed in duplicate.  ^∗^ *p* < 0.01 vs. NC (the unpaired Student’s *t*‐test).

**Figure:**
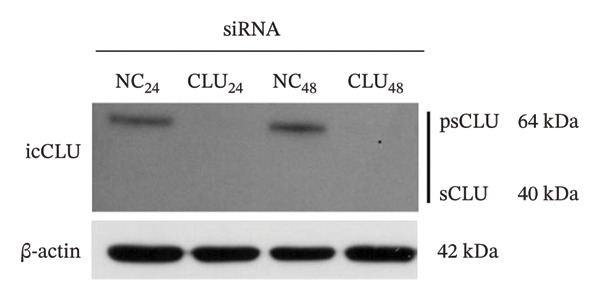
(a)

**Figure:**
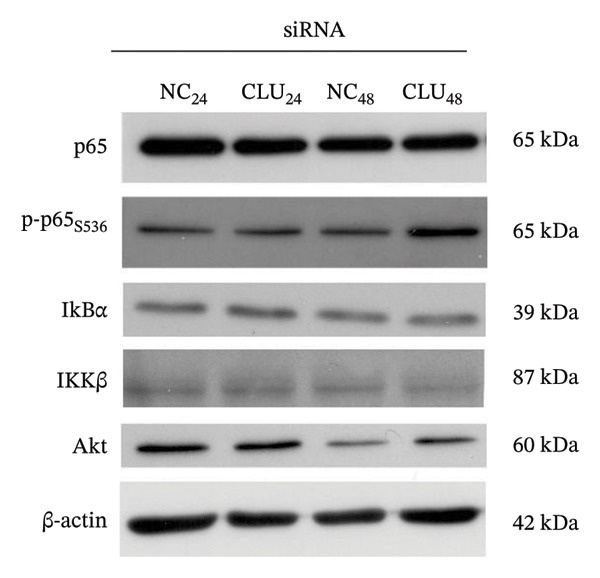
(b)

**Figure:**
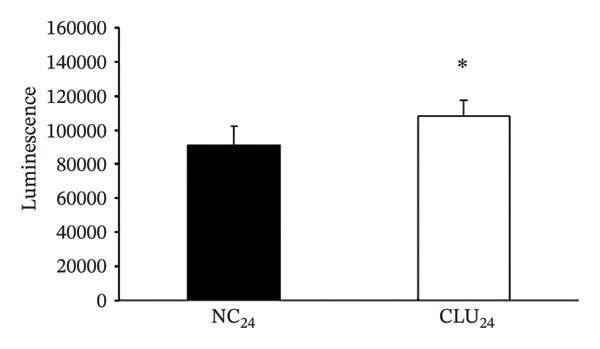
(c)

**Figure:**
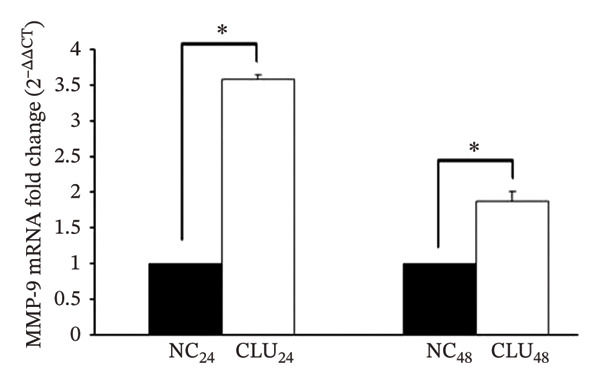
(d)

The correction does not affect the conclusions of the manuscript.

We apologize for this error.

