# Supplementary figures and images for: Clusterin Silencing in Prostate Cancer Induces Matrix Metalloproteinases by an NF-κB-Dependent Mechanism
Source: J Oncol. 2019 Dec 6;2019:4081624. doi: 10.1155/2019/4081624 (PMC6925831; doi:10.1155/2019/4081624)

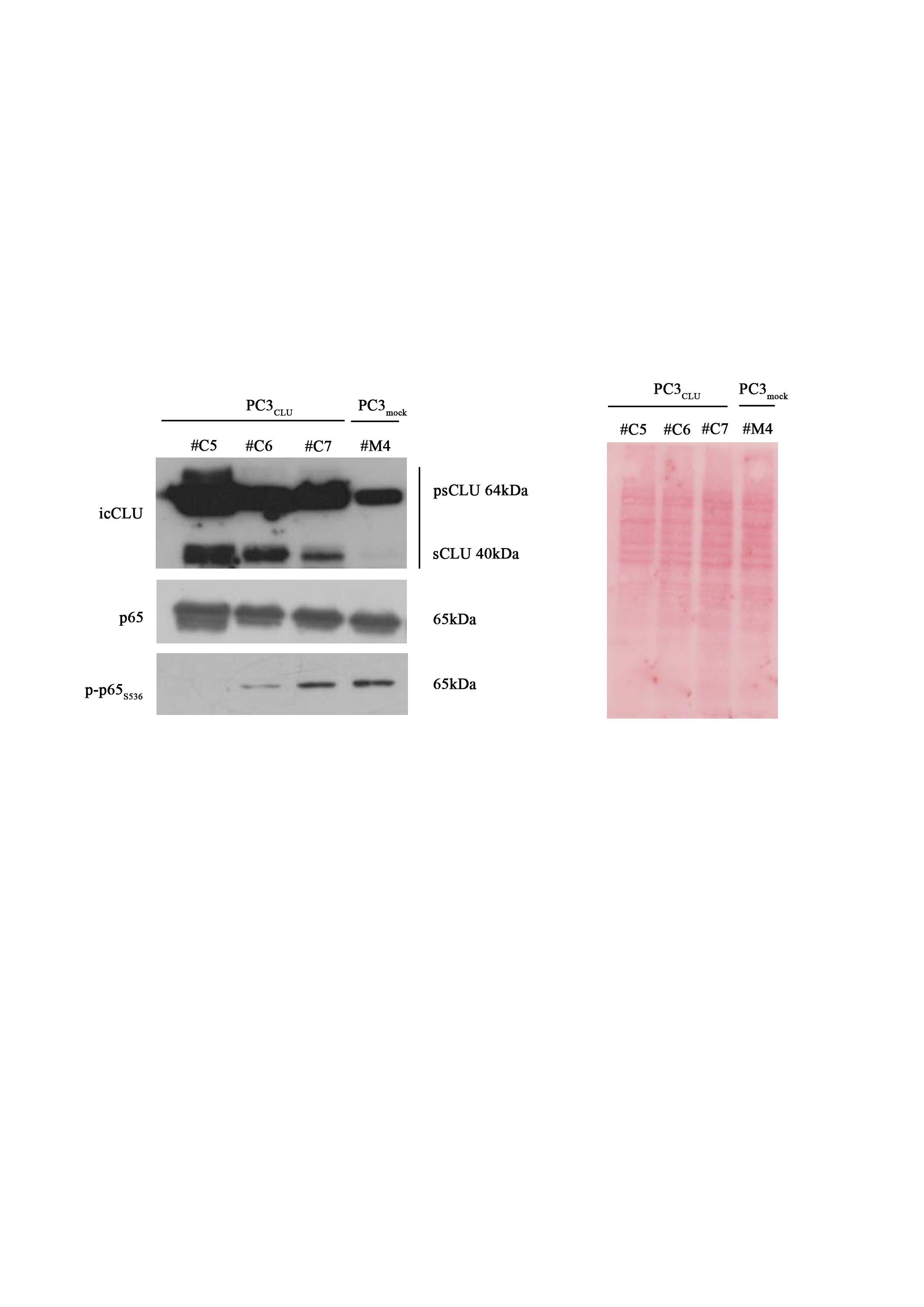

Supplement: Supplementary Materials — Figure S1: p65 expression and phosphorylation in additional PC3mock, PC3CLU, and p-p65S536 clones. Quantification of CLU, p65, and p-p65S536 protein by WB analysis in PC3CLU (namely, clones #C5, #C6, and #C7) and PC3mock (namely, clone #M4). Red Ponceau staining was used for loading and transfer control. icCLU, intracellular CLU; psCLU, uncleaved CLU precursor, 64 kDa; sCLU, cleaved mature CLU, 40 kDa. Figure S2: morphology, proliferation, and cell cycle analysis of PC3mock and PC3CLU clones. (a) Phase-contrast images of PC3 cells, PC3mock, and PC3CLU. Magnification: 20×. (b) Cellular proliferation was assessed in PC3mock (black square) and in PC3CLU (white square) clones at the indicated time after seeding by crystal violet assay. Error bars represent SD of the mean of three PC3mock and three PC3CLU clones. ∗p < 0.05 (the unpaired t-test vs. mock). (c) Cell cycle analysis was carried out in PC3mock and PC3CLU by FACS analysis. The graphs are representative of all the clones analyzed. The percentage of distribution of the cells in the cell cycle phases is reported in the table. Figure S3: expression of p65 in TRAMP and TRAMP/CLUKO prostate tissues. Immunohistochemical staining of p65 in prostate tissues of 12- and 24-week-old (left and right panels, respectively) TRAMP and TRAMP/CLUKO mice. Three animals for each experimental group were examined. Images magnification 40×. [file 4081624.f1.zip › 4081624.f1/Fig.S1.tif]

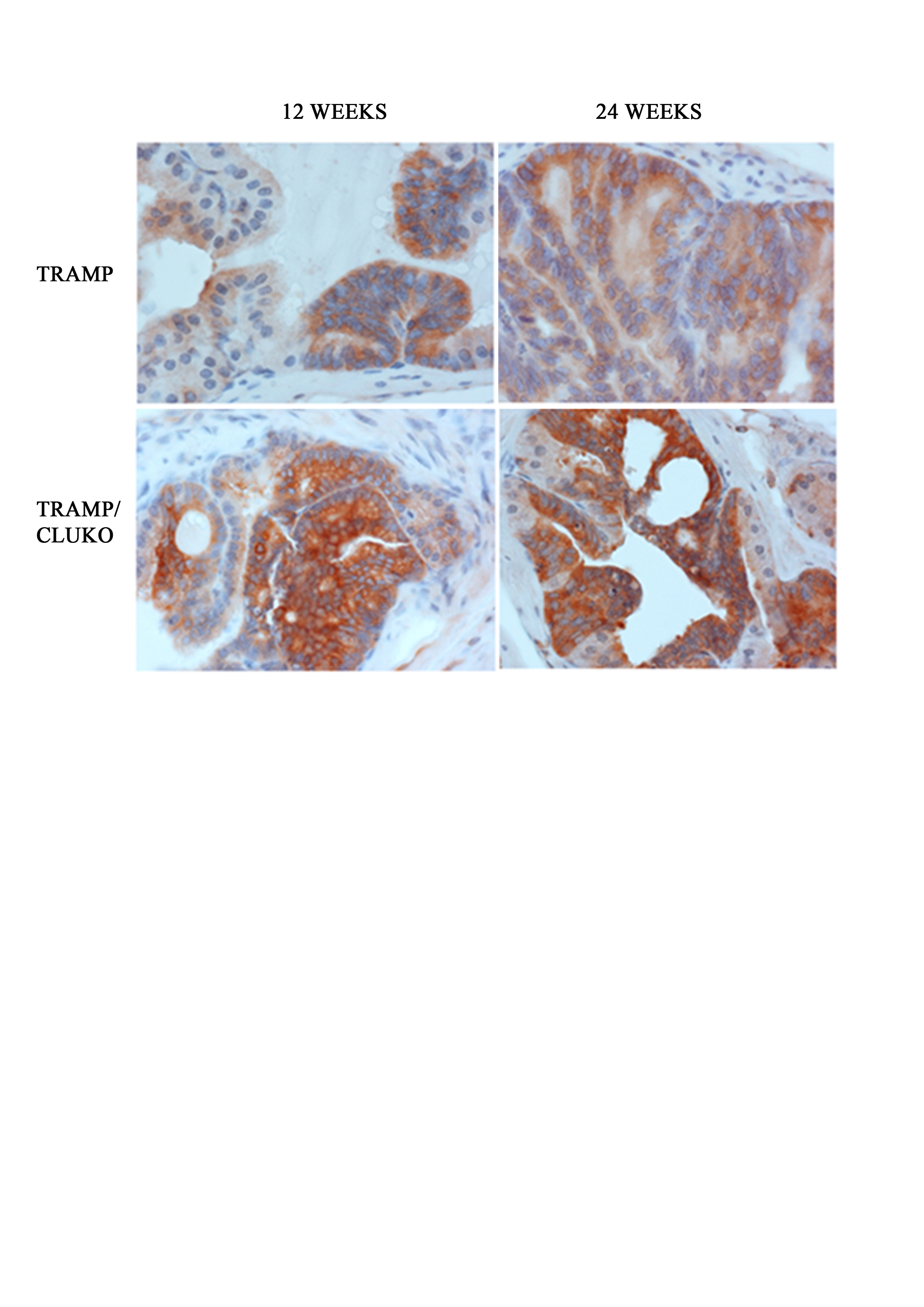

Supplement: Supplementary Materials — Figure S1: p65 expression and phosphorylation in additional PC3mock, PC3CLU, and p-p65S536 clones. Quantification of CLU, p65, and p-p65S536 protein by WB analysis in PC3CLU (namely, clones #C5, #C6, and #C7) and PC3mock (namely, clone #M4). Red Ponceau staining was used for loading and transfer control. icCLU, intracellular CLU; psCLU, uncleaved CLU precursor, 64 kDa; sCLU, cleaved mature CLU, 40 kDa. Figure S2: morphology, proliferation, and cell cycle analysis of PC3mock and PC3CLU clones. (a) Phase-contrast images of PC3 cells, PC3mock, and PC3CLU. Magnification: 20×. (b) Cellular proliferation was assessed in PC3mock (black square) and in PC3CLU (white square) clones at the indicated time after seeding by crystal violet assay. Error bars represent SD of the mean of three PC3mock and three PC3CLU clones. ∗p < 0.05 (the unpaired t-test vs. mock). (c) Cell cycle analysis was carried out in PC3mock and PC3CLU by FACS analysis. The graphs are representative of all the clones analyzed. The percentage of distribution of the cells in the cell cycle phases is reported in the table. Figure S3: expression of p65 in TRAMP and TRAMP/CLUKO prostate tissues. Immunohistochemical staining of p65 in prostate tissues of 12- and 24-week-old (left and right panels, respectively) TRAMP and TRAMP/CLUKO mice. Three animals for each experimental group were examined. Images magnification 40×. [file 4081624.f1.zip › 4081624.f1/FigS3 .tif]
